# Eight-Week Consumption of High-Sucrose Diet Has a Pro-Oxidant Effect and Alters the Function of the Salivary Glands of Rats

**DOI:** 10.3390/nu10101530

**Published:** 2018-10-17

**Authors:** Mateusz Maciejczyk, Jan Matczuk, Małgorzata Żendzian-Piotrowska, Wiesława Niklińska, Katarzyna Fejfer, Izabela Szarmach, Jerzy Robert Ładny, Izabela Zieniewska, Anna Zalewska

**Affiliations:** 1 Department of Physiology, Medical University of Bialystok, 2c Mickiewicza Street, 15-233 Bialystok, Poland; mat.maciejczyk@gmail.com; 2 County Veterinary Inspection, Zwycięstwa 26b Street, 15-959 Bialystok, Poland; jmatczuk@yahoo.com; 3 Department of Hygiene, Epidemiology and Ergonomics, Medical University of Bialystok, 15-233 Bialystok, Poland; mzpiotrowska@gmail.com; 4 Department of Histology and Embryology, Medical University of Bialystok, Waszyngtona 13 Street, 15-233 Bialystok, Poland; wieslawa.niklinska@umb.edu.pl; 5 Department of Conservative Dentistry, Medical University of Bialystok, Sklodowska 24a Street, 15-274 Bialystok, Poland; ksawicka1@wp.pl (K.F.); izazieniewska@gmail.com (I.Z.); 6 Department of Orthodontics, Medical University of Bialystok, Waszyngtona 15a Street, 15-274 Bialystok, Poland; orthod@umb.edu.pl; 7 Department of Emergency Medicine and Disasters, Medical University of Bialystok, Szpitalna 37 Street, 15-767 Bialystok, Poland; ladnyjr@wp.pl

**Keywords:** sucrose, salivary glands, oxidative stress, salivary antioxidants, insulin resistance

## Abstract

A high-sucrose diet (HSD) is widely known for its cariogenic effects and promotion of obesity, insulin resistance, type 2 diabetes, and cancer. However, the impact of the HSD diet on the salivary gland function as well as the level of salivary oxidative stress is still unknown and requires evaluation. Our study is the first to determine both redox balance and oxidative injury in the parotid and submandibular glands of rats fed the HSD diet compared to the control group. We have demonstrated that uric acid concentration and the activity of superoxide dismutase and peroxidase varied significantly in both the submandibular and parotid glands of HSD rats vs. the control group. However, enhanced oxidative damage to proteins, lipids, and DNA (increase in advanced glycation end products, advanced oxidation protein products, 4-hydroxynonenal, and 8-hydroxy-2’-deoxyguanosine) was observed only in the parotid glands of HSD rats. Moreover, the HSD diet also reduced the total protein content and amylase activity in both types of salivary glands and decreased the stimulated salivary flow rate. To sum up, an HSD diet reduces salivary gland function and disturbs the redox balance of the parotid as well as submandibular salivary glands. However, the parotid glands are more vulnerable to both antioxidant disturbances and oxidative damage.

## 1. Introduction

Free sugars (glucose, fructose) and sucrose are the main substrates used for energy production, which facilitates a positive energy balance [1]. Sustaining the energy balance is key to maintaining a proper body weight (for adults: 25 kg/m^2^ > BMI ≥ 18.5 kg/m^2^) and ensuring optimal nutrient consumption [2]. It has been proven that the worldwide intake of free sugars varies by age and place of residence and shows an increasing tendency from year to year, which is alarming. WHO strongly recommends reducing daily intake of free sugars for both adults and children to less than 10% of total energy intake. According to WHO’s conditional recommendation, further reduction of free sugar intake to a level below 5% per day would provide additional health benefits [3].

Chronic intake of a free sugar-rich diet increases overall energy intake, reduces consumption of food containing more nutritionally adequate calories, and contributes to malnutrition, becoming overweight, obesity, insulin resistance, type 2 diabetes, elevation coronary artery disease, and even cancer [4,5,6,7,8]. Although the pathogenesis of the above disturbances is generally complex and diverse, it has been demonstrated that excess production of reactive oxygen species (ROS) in the course of chronic high-sucrose consumption may be one of the factors facilitating initiation and progression of these metabolic disorders [9]. In these conditions, insufficiency of the antioxidant systems to combat excessive ROS generation leads to oxidative stress (OS) and redox abnormalities. OS is a condition accompanied by oxidative damage to bio-macromolecules such as proteins, lipids, and nucleic acids, which disturbs cellular metabolism and its regulation [10,11]. There are numerous reports on the increased oxidative damage to human or experimental animal plasma, liver, skeletal muscles [4,5,7,8], and heart [6] in the course of a high-sucrose diet.

Chronic high-sucrose consumption is also a key factor in the development of pathological changes in the oral homeostasis in a form of increased dental decay [12,13]. It should be additionally emphasized that ingestion of a high-sucrose diet has been linked to dysfunction of parotid glands [14,15]. Processes leading to dysfunction of salivary glands during the high-sucrose diet have not been identified yet. However, the issue still deserves attention. Saliva produced by the salivary glands plays the most important role in oral cavity homeostasis since it creates an environment suitable for the oral cavity and cleansing of both the mucous membrane and the teeth. The salivary proteins and enzymes serve a moisturizing function, which facilitates articulation and swallowing. They also regulate pH, protect teeth from decay, and are responsible for preliminary food digestion. Moreover, saliva participates in both specific and non-specific immune defense mechanisms [16,17] and demonstrates effective anti-oxidative properties, which constitutes the first line of defense of the gastrointestinal tract against ROS [18].

The impact of a high-sucrose diet on salivary secretion, antioxidant barrier, and oxidative stress in the salivary glands is still unknown. Therefore, it is important to explain whether the chronic intake of high sucrose levels results in a disturbed redox balance in the salivary glands. Our goal was also to search for a relationship between oxidative stress and salivary gland function in the course of a high-sucrose diet.

## 2. Materials and Methods

After seven days of an adaptation period, male Wistar rats (*n* = 20, initial age: six weeks) were randomly divided into two groups, as shown below.

normal chow (C, *n* = 10),

high sucrose-fed group (HSD, *n* = 10).

All the animals were kept in the conditions of 12:12 h light/dark cycle and controlled temperature (20–21 °C). During the entire experiment, the rats stayed separately in standard cages with unlimited access to food and drinking water.

All of the study procedures were approved by the Committee for Ethical Treatment of Animals at the Medical University in Bialystok, Poland (protocol number 89/2015, 2015/109).

For eight consecutive weeks of the experiment, the control group was fed a control diet (Research Diets, Inc., New Brunswick, NJ, USA, D12450K) consisting of 70 kcal% carbohydrates (sucrose 0 kcal, cornstarch 2200 kcal, maltodextrin 10,600 kcal), 20 kcal% protein, and 10 kcal% fat. The HSD group was fed a high-sucrose diet (Research Diets, Inc., New Brunswick, NJ, USA; D12450B) composed of 70 kcal% carbohydrates (sucrose 1400 kcal, cornstarch 1260 kcal, maltodextrin 10 140 kcal), 20 kcal% protein, and 10 kcal% fat [19]. The caloricity of both diets was the same and amounted to 3.85 kcal/g.

The food consumption was measured every two days and fluid consumption was monitored weekly. The rats were weighted on the first day of the experiment and then weekly and immediately prior to sacrifice.

After eight weeks of treatment, the rats were fasted for 12 h and anesthetized with phenobarbital (80 mg/kg body weight, intraperitoneally). The rats were placed in a supine position on the heated couch (37 °C) and then had their tail blood collected to measure blood glucose concentration (Accu Check, Roche). Next, whole unstimulated saliva was collected from the oral cavity with the pre-weighted cotton ball inserted under the tongue and bilaterally medial to the teeth and oral mucosa for 15 min [20,21]. Whole stimulated saliva was being collected 5 min after the injection of pilocarpine nitrate (5 mg/kg body weight, intraperitoneally, Sigma Chemical Co, St. Louis, MO, USA) for 5 min [20,21]. The volume of unstimulated/stimulated saliva was evaluated by subtracting the initial weight of cotton balls from their final weight. One mg of the collected saliva was considered to be 1 µL [20,21].

Subsequently, whole blood was collected from the abdominal aorta into heparinized tubes and centrifuged (5 min, 4 °C, 3000× *g*, MPW 351, MPW Med. Instruments, Warsaw, Poland). Then the antioxidant butyl-hydroxytoluene (BHT, 10 µL 0.5 M BHT in acetonitrile per 1 mL of the plasma Sigma-Aldrich, Steinheim, Germany) was added to the collected plasma samples. The plasma was pre-cooled in liquid nitrogen and stored at −80 °C until it was analyzed [22].

After blood collection, the parotid and submandibular glands of rats were removed. Those intended for biochemical determination were weighted, freeze-clamped with aluminum tongs, frozen in liquid nitrogen, and stored at −80 °C until biochemical experiments were conducted. The right salivary glands were intended for histological analysis and were fixed with 10% buffered formalin solution.

On the day of biochemical assays, the salivary glands were thawed (4 °C), weighted, and divided into small pieces. Then they were diluted in PBS (1:10) and the fragments of salivary glands intended for the determination of carbonyl groups were diluted in 50 mM phosphate buffer (1:10). Both PBS and phosphate buffer were enriched with BHT (10 µL 0.5 M BHT/1 mL buffer) and a protease inhibitor (1 tablet/10 mL buffer, Complete Mini Roche, France). Next, salivary glands were homogenized (Omni TH, Omni International, Kennesaw, GA, USA, on ice, 5 × 20 s), which was followed by their sonification (1800 J/sample, 3 × 20 s, on ice; UP 400S, Hielscher, Teltow, Germany). The supernatants were analyzed immediately after centrifugation (for 20 min, 4 °C, 5000× *g*, MPW Med Instruments, Warsaw, Poland).

The insulin concentration was analyzed using the commercial ELISA kit (Shibayagi Co., Gunma, Japan; Cell Biolabs), according to the manufacturer’s instructions. The insulin sensitivity was evaluated by using the HOMA-IR index (homeostasis model assessment of insulin resistance = fasting insulin (U/mL) × fasting glucose (mM)/22.5) [23].

### 2.1. Biochemical Analysis

The performed analysis included the total protein, salivary amylase, non-enzymatic and enzymatic antioxidants (uric acid (UA), catalase (CAT, EC 1.11.1.6), Cu-Zn superoxide dismutase (SOD, EC 1.15.1.1), salivary peroxidase (Px, EC 1.11.1.7), glutathione peroxidase (GPx, EC 1.11.1.9), the total antioxidant status (TAS)), oxidative damage products [advanced glycation end products (AGE), advanced oxidation protein products (AOPP), protein carbonyls (PC), 4-hydroxynonenal protein adducts (4-HNE), 8-isoprostanes (8-isop), 8-hydroxy-2’-deoxyguanosine (8-OHdG), and total oxidant status (TOS) as well as interleukin-1β (IL-1 β), NADPH oxidase (EC 1.6.3.1), and the ROS production rate. All the assays were performed in homogenates of salivary glands as well as plasma in duplicate samples (unless marked otherwise). The absorbance was measured by using the Infinite M200 PRO Multimode Microplate Reader, Tecan. The results were standardized to 100 mg of the total protein.

### 2.2. Salivary and Plasma Antioxidants

The concentration of UA was measured colorimetrically by using the commercial kit QuantiChrom^TM^ Uric Acid DIUA-250 (BioAssay Systems, Harward, CA, USA) in accordance with the manufacturer’s instructions. In this assay, 2,4,6-tripyridyl-s-triazine (TPTZ) forms a blue complex with iron ions in the presence of UA. The intensity of the resulting complex was measured at 490 nm wavelength.

The activity of CAT was estimated in triplicate samples by measuring the decomposition rate of hydrogen peroxide (H_2_O_2_) at 240 nm wavelength [24]. One unit of CAT activity was defined as the amount of enzyme that decomposes 1 mmol H_2_O_2_ for 1 min.

The activity of SOD was assayed colorimetrically by measuring the cytosolic activity of SOD and by inhibiting the oxidation of adrenaline to adrenochrome at 480 nm [25]. It was assumed that one unit of SOD activity inhibits the oxidation of adrenaline by 50%.

The activity of Px was determined colorimetrically at a 412 nm wavelength based on the reduction of 5,5′-dithiobis-(2-nitrobenzoic acid) to thionitrobenzene acid that reacts with hypothiocyanite—the product of potassium thiocyanate oxidation by Px [26]. A decrease in the absorbance of thionitrobenzene acid was measured five times at 30-s intervals.

The activity of GPx was measured by the Paglia and Valentine colorimetric method [27]. This assay is based on the oxidation of reduced glutathione (GSH) and the reduction of organic peroxides by GPx in the presence of NADPH and glutathione reductase (GR). One unit of GPx activity was assumed to catalyze the oxidation of 1 μmol of NADPH for 1 min.

The concentration of TAS was analyzed in triplicate samples by using a 2,2-azinobis-3-ethylbenzothiazoline-6-sulfonic acid radical cation (ABTS^*+^) [28]. Changes in the absorbance of the ABTS^*+^ were determined at a 660 nm wavelength and TAS was calculated from the calibration curve for Trolox (6-hydroxy-2,5,7,8-tetramethylchroman-2-carboxylic acid).

The concentration of total protein was determined via the colorimetric bicinchoninic acid (BCA) method using the commercial kit Thermo Scientific PIERCE BCA Protein Assay (Rockford, IL, USA) with bovine serum albumin (BSA) as a standard.

### 2.3. Salivary and Plasma Oxidative Damage Products

The content of AGE was assayed fluorimetrically by measuring AGE-specific fluorescence (350 nm/440 nm) [29]. For AGE determination in plasma, plasma samples were diluted 1:5 (*v*/*v*) in phosphate buffered saline (PBS, pH 7.2) [30].

The concentration of AOPP was determined colorimetrically by measuring the oxidative capacity of the iodine ion at 340 nm [29]. For AOPP determination in plasma, plasma samples were diluted in PBS (pH 7.2) 1:5 (*v*/*v*) [30].

The concentration of PC was analyzed by 2,4-dinitrophenylhydrazine (2,4-DNPH) [31]. The principle of the method is based on the reaction of 2,4-DNPH with carbonyl groups in the oxidatively damaged proteins, which results in a hydrazone determined colorimetrically at a 355 nm wavelength. PC was calculated by using an absorption coefficient for 2,4-DNPH = 22,000 M^−1^ cm^−1^.

The concentrations of 4-HNE, 8-isoP, and 8-OHdG were determined with an ELISA test using commercial kits (4-HNE protein adduct ELISA kit, Cell Biolabs, Inc., San Diego, CA, USA, 8-isoprostane ELISA Kit, Cayman Chemicals, Ann Arbor, MI, USA, 8-OHdG ELISA kit, USCN Life Science, Wuhan, China) in accordance with the manufacturer’s instructions.

The concentration of TOS was estimated in triplicate samples, according to the method described by Erel [32]. This assay is based on the oxidation of the ions Fe^2+^ to Fe^3+^ in the presence of the oxidants contained in the sample. The absorbance was measured bi-chromatically at a 560/800 nm wavelength. The oxidative stress index (OSI) was calculated from the formula: OSI = TOS/TAS × 100 [33].

### 2.4. Salivary and Plasma Inflammation and ROS Production

The concentration of IL-1β was estimated by the ELISA method using ready-made kits (R & D Systems, Canada, Minneapolis, MN, USA) in accordance to the manufacturer’s instructions. The absorbance was analyzed at 450 nm.

The activity of NADPH oxidase was measured by a luminescence assay using lucigenin as a luminophore [34]. One unit of NADPH oxidase activity was defined as the amount of enzyme required to release 1 nmol of superoxide anion for 1 min.

The rate of ROS production was assayed using 2,7-dichlorodihydrofluorescein diacetate (DCFH-DA), which is oxidized to the fluorescent 2,7-dichlorodihydrofluorescein (DCFH) by oxygen free radicals [35]. The fluorescence was measured at 488/525 nm.

### 2.5. Histological Examination

The left salivary glands were processed for paraffin embedding. Next, five-micron sections were prepared and stained with hematoxylin-eosin. The sections were analyzed under a light microscope (OLYMPUS BX 51, Olympus America Inc., Center Valley, PA, USA) at 40× and 60× magnification by one histologist (Wiesława Niklińska)

### 2.6. Statistical Analysis

The data was analyzed by using the program Statistica 10.0 (StatSoft, Krakow, Poland). The Kolmogorov-Smirnov test showed no normal distribution of the obtained results, which was the reason for using non-parametric methods. The assessment of differences in the distribution of quantitative variables between the two groups was conducted by using the U Mann Whitney test. The distribution of values of individual quantitative parameters was presented in the form of medians (minimum-maximum). The Spearman’s correlation coefficient was used to study the associations between the examined parameters. The statistical significance was defined as *p* ≤ 0.05.

## 3. Results

### 3.1. General Characteristics

Food consumption, energy intake, and the weight of submandibular glands were comparable between HSD rats and the control group (Table 1). However, the study demonstrated that the eight-week high-sucrose diet affects the final body weight and the parotid glands’ weight. The medians of the final body weight and the parotid glands’ weight of HSD rats were significantly raised when compared to the controls (*p* = 0.005 and *p* = 0.05, respectively). The high-sucrose diet altered glucose homeostasis because fasting glucose and insulin concentration as well as HOMA-IR were significantly increased compared to the control rats (*p* = 0.0008, *p* = 0.02, and *p* < 0.0001, respectively) (Table 1).

**Figure 1:**
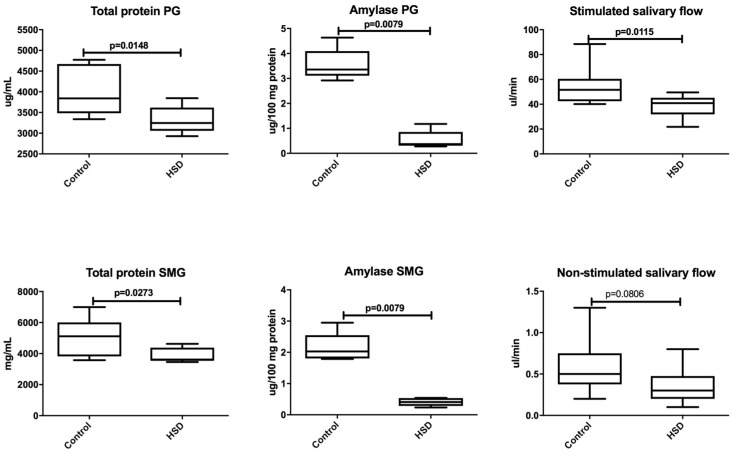
The effect of high-sucrose diet (HSD) on total protein, amylase activity, and non-stimulated and stimulated salivary flow rate. Abbreviations: HSD, high-sucrose diet, PG, parotid gland, SMG, submandibular gland.

High-sucrose feeding influenced protein concentration and amylase activity in both salivary glands of HSD rats (Figure 1). Total protein concentrations in both: the parotid (↓ 16%) and submandibular glands (↓ 36%) of HSD rats were significantly decreased when compared to the controls (*p* = 0.01 and *p* = 0.02, respectively). As presented in Figure 1, amylase activity in the tissue of parotid (↓ 87.8%) and submandibular (↓ 82%) glands of HSD rats decreased considerably in comparison to the control rats’ glands (*p* = 0.008 and *p* = 0.008, respectively). 

Figure 1 shows that high-sucrose feeding had no influence on the non-stimulated secretory function of salivary glands in HSD rats when compared to the control group. Contrary to unstimulated salivary flow, stimulated saliva secretion in HSD rats was significantly decreased (↓ 34%) when compared to the control rats (*p* = 0.01).

### 3.2. Plasma Antioxidants and Oxidative Modification Products

**Figure 2:**
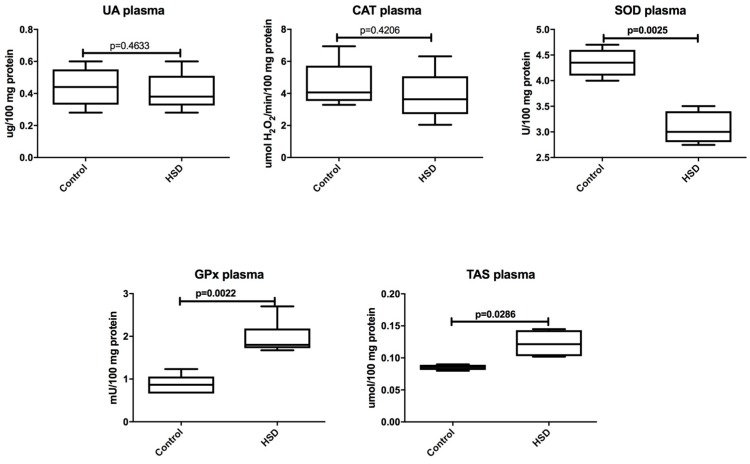
The effect of a high-sucrose diet (HSD) on the plasma antioxidant defense. Abbreviations: CAT, catalase, GPx, glutathione peroxidase, Px, salivary peroxidase, SOD, superoxide dismutase-1, TAS, total antioxidant status, UA, uric acid.

Plasma UA concentration and CAT activity were similar for HSD and the control group. In the plasma of HSD rats, SOD activity was decreased (*p* = 0.002) and GPx activity (*p* = 0.002) as well as TAS concentration were increased (*p* = 0.03) when compared to the control group (Figure 2).

**Figure 3:**
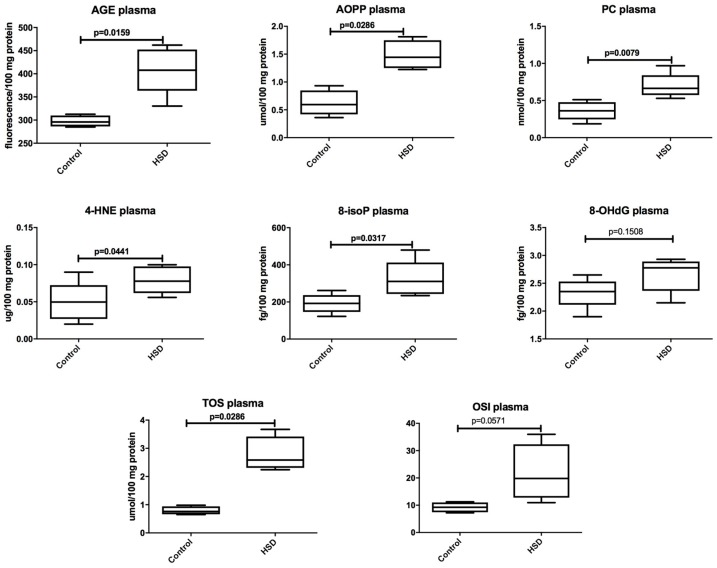
The effect of a high-sucrose diet (HSD) on plasma oxidative damage. Abbreviations: 4-HNE, 4-hydroxynonenal protein adducts, 8-isoP, 8-isoprostanes, 8-OHdG, 8-hydroxy-2′-deoxyguanosine, AGE, advanced glycation end products, AOPP, advanced oxidation protein products, OSI, oxidative stress index, PC, protein carbonyls, TOS, total oxidant status.

The plasma concentrations of AGE (*p* = 0.01), AOPP (*p* = 0.03), PC (*p* = 0.008), 4-HNE protein adducts (*p* = 0.04), 8-isoP (*p* = 0.03), and TOS (*p* = 0.03) were significantly higher in the plasma of the HSD rats when compared to the controls. The plasma concentration of OSI and 8-OHdG were similar in both groups (Figure 3).

### 3.3. Plasma Inflammation and ROS Production

**Figure 4:**
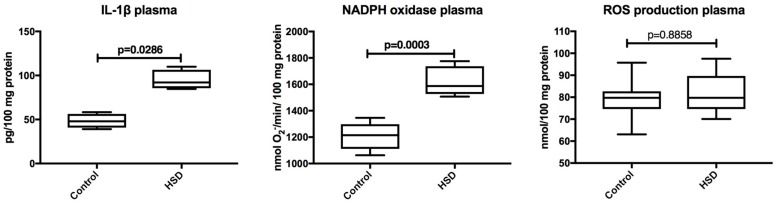
The effect of a high-sucrose diet (HSD) on plasma inflammation and reactive oxygen species (ROS) production. Abbreviations: IL-1β, interleukin-1β, ROS, reactive oxygen species.

Plasma IL-1β level and NADPH oxidase activity were significantly higher in HSD rats when compared to the controls (*p* = 0.03, *p* = 0.0003, respectivetly). The rate of ROS production did not statistically differ between the study and the control animals (Figure 4).

### 3.4. Salivary Glands Antioxidants and Oxidative Modification Products

**Figure 5:**
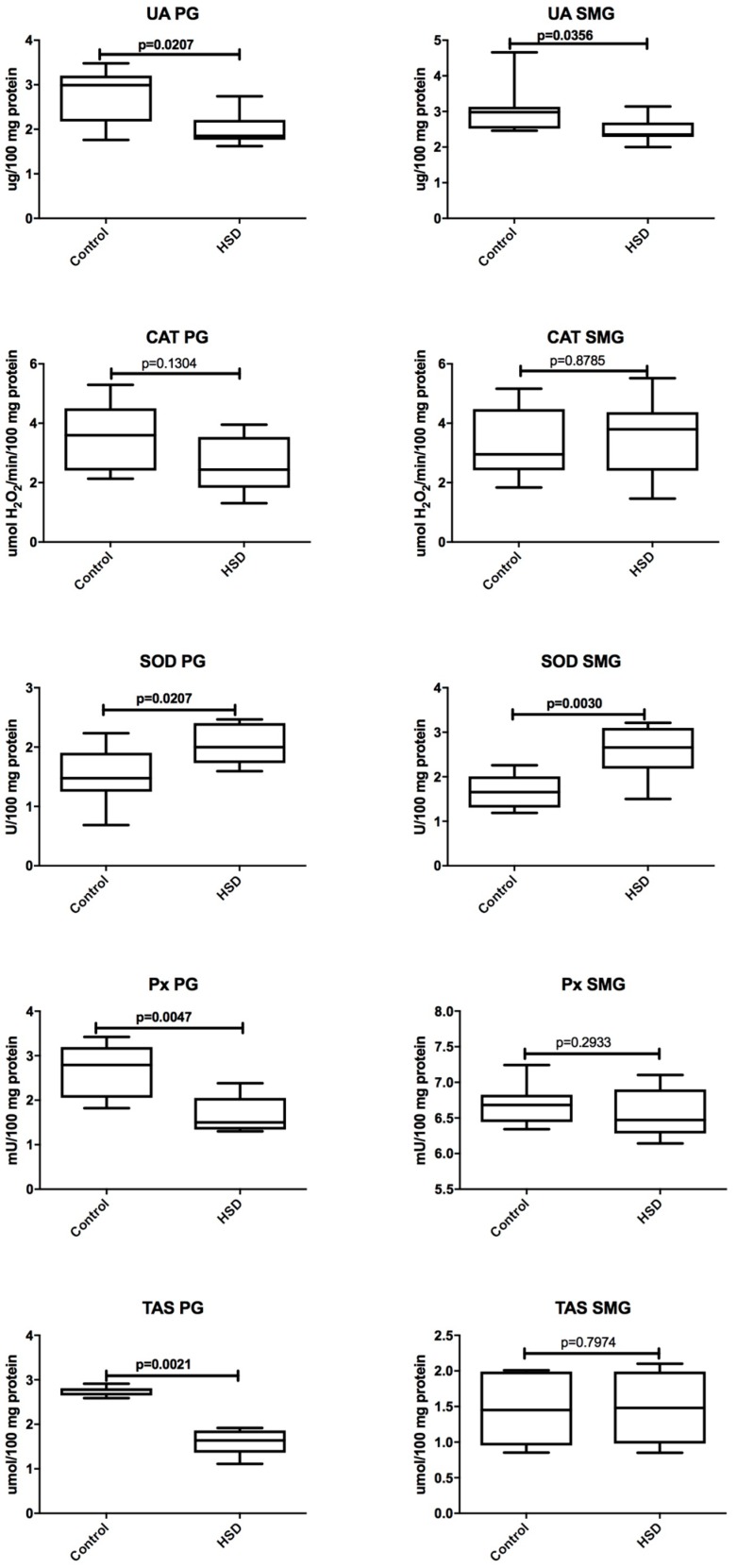
The effect of a high-sucrose diet (HSD) on the salivary glands’ antioxidant defense. Abbreviations: CAT, catalase, PG, parotid glands, Px, salivary peroxidase, SOD, superoxide dismutase-1, SMG, submandibular glands, TAS, total antioxidant status, UA, uric acid.

The effect of eight-week high-sucrose diet on the parotid gland antioxidant defense is presented in Figure 5. We noted that only SOD activity in the parotid glands of HSD rats was significantly increased when compared to the control rats (↑ 35%, *p* = 0.02). The concentration of UA (↓ 39%, *p* = 0.02) and TAS (↓ 58%, *p* = 0.002) as well as Px activity (↓ 46%, *p* = 0.004) in the parotid glands of HSD rats were considerably lower than in the control rats while CAT activity in the parotid glands of the HSD group did not differ from the control group.

We observed that the concentration of UA (↓ 22%, *p* = 0.03) decreased but the SOD activity (↑ 60%, *p* = 0.003) increased in the submandibular glands of HSD rats when compared to the control group. CAT and Px activity as well as TAS concentration were similar in the submandibular glands of the HSD and the control groups (Figure 5).

**Figure 6:**
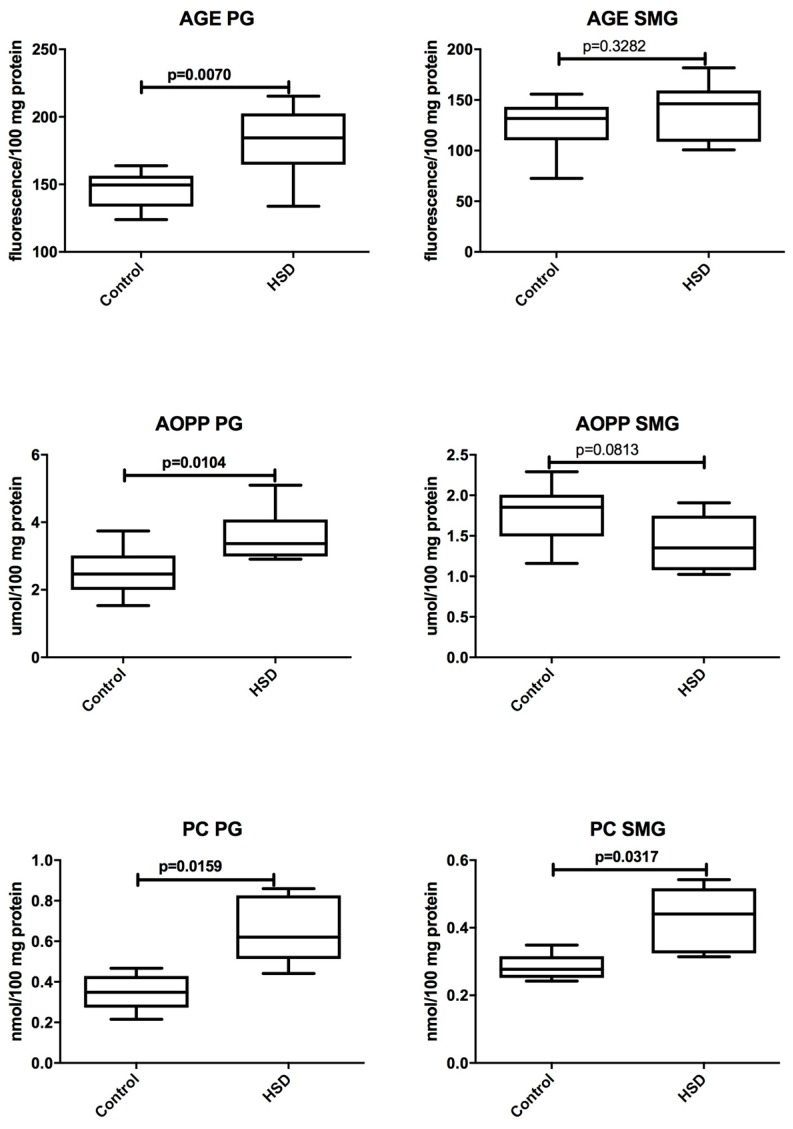
The effect of a high-sucrose diet (HSD) on protein oxidative damage. Abbreviations: AGE, advanced glycation end products, AOPP, advanced oxidation protein products, PC, protein carbonyls, PG, parotid glands, SMG, submandibular glands.

**Figure 7:**
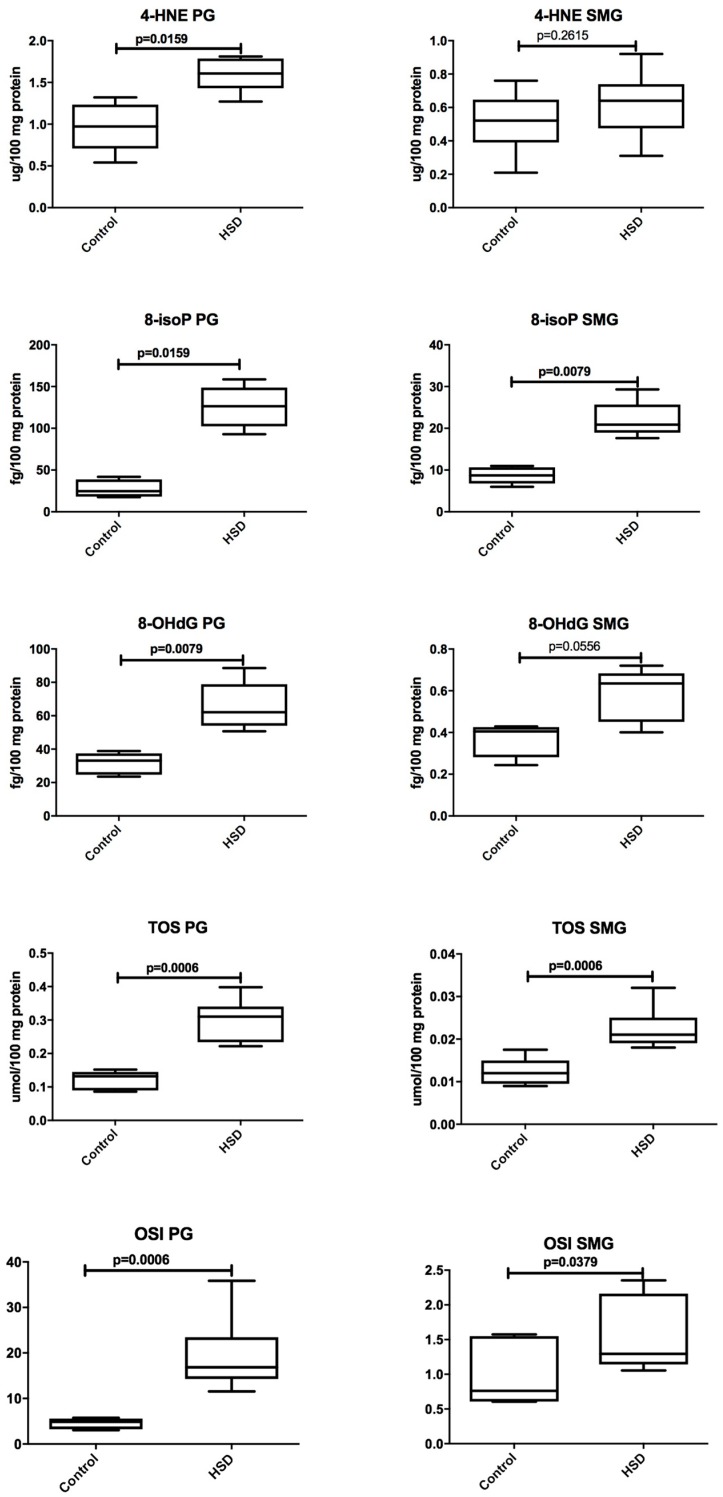
The effect of a high-sucrose diet (HSD) on lipid and DNA oxidative damage. Abbreviations: 4-HNE, 4-hydroxynonenal protein adducts, 8-isoP, 8-isoprostanes, 8-OHdG, 8-hydroxy-2’-deoxyguanosine, OSI, oxidative stress index, PG, parotid glands, SMG, submandibular glands, TOS, total oxidant status.

Figure 6 and Figure 7 present changes in the oxidative stress parameters of the parotid glands in the course of high-sucrose feeding. We observed significant increases in all oxidative stress parameters in the parotid glands of the HSD group when compared to the controls: AGE (↑ 23%, *p* = 0.007), AOPP (↑ 36%, *p* = 0.01), PC (↑ 82%, *p* = 0.01), 8-isoP (↑ 410%, *p* = 0.01), 4-HNE protein adducts (↑ 65%, *p* = 0.01), 8-OHdG (↑ 87%, *p* = 0.007), TOS (↑ 138%, *p* = 0.0006), and OSI (↑ 245%, *p* = 0.0006). Of all the tested the parameters, only the concentrations of PC (↑ 57%, *p* = 0.03), 8-isoP (↑ 139%, *p* = 0.007), TOS (↑ 100%, *p* = 0.0006), and OSI (↑ 70%, *p* = 0.04) were significantly increased in the submandibular glands of HSD rats when compared to the controls. AGE, AOPP, and 4-HNE protein adducts and 8-OHdG in the submandibular glands of the HSD group did not differ from the control group (Figure 6 and Figure 7).

### 3.5. Salivary Glands Inflammation and ROS Production

**Figure 8:**
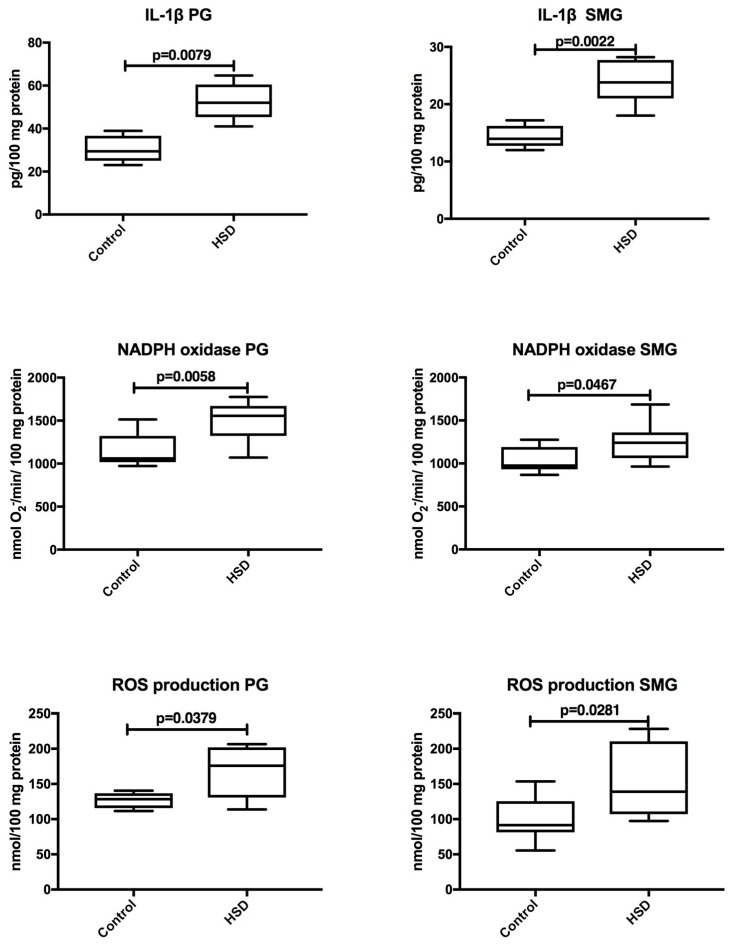
The effect of a high-sucrose diet (HSD) on salivary glands inflammation and reactive oxygen species (ROS) production. Abbreviations: IL-1β, interleukin-1β, PG, parotid glands, SMG, submandibular glands.

The level of IL-1β, activity of NADPH oxidase, and the rate of ROS production were significantly enhanced in both parotid (*p* = 0.008, *p* = 0.006, *p* = 0.04, respectively) and submandibular glands (*p* = 0.002, *p* = 0.05, *p* = 0.03, respectively) of HSD rats when compared to the controls (Figure 8). 

### 3.6. Parotid Glands vs. Submandibular Glands

Total protein concentration and peroxidase activity were significantly higher in the submandibular glands vs. parotid glands of the control rats (*p* = 0.05 and *p* = 0.0002, respectively). However, the concentrations of TAS (*p* = 0.002), AOPP (*p* = 0.02), 8-isoP (*p* = 0.02), 4-HNE protein adducts (*p* = 0.02), 8-OHdG (*p* = 0.008), TOS (*p* = 0.0006), OSI (*p* = 0.0006), and IL-1β (*p* = 0.004) were considerably higher in the parotid than in the submandibular glands of the control group (Table 2).

Total protein (*p* = 0.02) and UA (*p* = 0.01) concentrations as well as the activity of SOD (*p* = 0.04) and Px (*p* = 0.0002) were significantly increased in the submandibular glands when compared to the parotid glands of the HSD group. The levels of AGE (*p* = 0.01), AOPP (*p* = 0.0007), PC (*p* = 0.03), 8-isoP (*p* = 0.008), 4-HNE protein adducts (*p* = 0.004), 8-OHdG (*p* = 0.008), OSI (*p* = 0.0006), and IL-1β (*p* = 0.004) as well as the activity of NADPH oxidase (*p* = 0.049) and the rate of the ROS production (*p* = 0.041) were significantly higher in the parotid compared to the submandibular glands of HSD rats (Table 3).

### 3.7. Correlations

The Spearman’s Correlation Coefficient showed that the level of 4-HNE protein adducts (*p* = 0.03, *r* = −0.43) in parotid glands of HSD rats was negatively correlated with a stimulated salivary flow rate. In the parotid glands of HSD rats, positive correlations were noted between PC (*p* = 0.03, *r* = 0.47) as well as AOPP (*p* = 0.02, *r* = 0.51) and plasma HOMA-IR. We also observed a positive correlation between plasma insulin concentration and PC (*p* = 0.03, *r* = 0.46) in submandibular glands of the HSD group.

### 3.8. Histological Analysis

**Figure 9:**
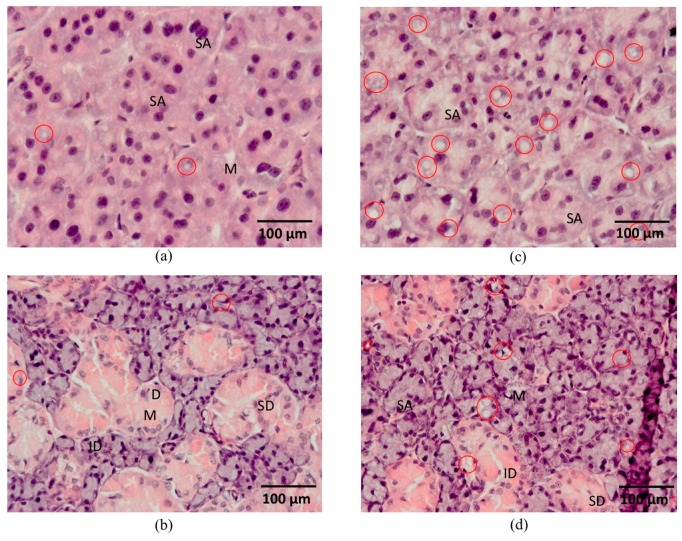
The influence of a high-sucrose diet (HSD) on histological observation of the salivary glands. Abbreviations: (**a**) parotid gland of the control rats, (**b**) submandibular gland of the control rats, (**c**) parotid gland of rats fed a high-sucrose diet (HSD), (**d**) submandibular gland of rats fed a high-sucrose diet (HSD), D, demilune, ID, intercalated ducts, M, mucous acini, SA, serous acini, SD, striated ducts. Red circles indicate vacuoles.

In the salivary glands of both groups of rats, we observed a typical lobular structure that was similar in the acini and duct architecture. In the microscopic image, visible severe diffuse vacuolization in the cytoplasm of parotid gland acini was noted in the HSD group and vacuoles in the cytoplasm of acini of the parotid glands of control rats were very few in number and small in size (Figure 9).

Similar to parotid glands of HSD rats but less numerous, vacuoles were seen in the cytoplasm of serous cells of the submandibular glands of HSD rats. Cytoplasmic vacuolization was not observed in the submandibular glands of the controls (Figure 9).

## 4. Discussion

The presented study evaluated changes in the antioxidant level, oxidative damage, and secretory function of the salivary glands of rats fed a high-sucrose diet. Generally, in the course of eight weeks of the diet, the antioxidant barrier of salivary glands was significantly affected. We demonstrated that both the parotid and submandibular glands of HSD rats are exposed to an oxidative stress condition. The stimulated salivary flow was significantly decreased and salivary amylase activity was statistically reduced in both types of glands of HSD rats compared to the control group.

A chronic high free-sugar diet results in obesity, hyperinsulinemia, hyperglycemia, insulin resistance, and general oxidative stress [6,36,37,38], which was confirmed by the results of our study. We noticed that an eight-week high-sucrose feeding significantly increased body weight and affected blood glucose homeostasis assessed on the basis of higher concentrations of blood insulin and fasting glucose in the HSD group rats compared to the control rats. Chronic high-sucrose feeding decreased whole-body insulin sensitivity, which was observed as a significant increase in the HOMA-IR index. According to the guidelines on diagnosing insulin-resistance [39,40], the latter result proves insulin resistance developed in the HSD group rats. Lastly, a considerable increase in the plasma concentration of almost all the examined oxidative stress markers (TOS, AGE, AOPP, PC, 8-isoP, 4-HNE protein adducts) confirmed a shift of redox balance towards the oxidative status in the HSD rats vs. the control group. The high-percentage increase in plasma 8-isoP is also noteworthy. Plasma 8-isoP promotes endothelin production, enhances platelet aggregation, and reduces the vascular effect of NO, which may consequently result in cardiovascular diseases [41].

It should be mentioned that a high-sucrose diet may contribute to the development of prooxidative milieu in several possible aspects. First of all, an excess glucose supply in the respiratory chain increases dramatically during high-sucrose feeding, which leads to the formation of excessive quantities of superoxide anions and other free radical species [5]. A high-sucrose diet has been proven to cause lipogenic effects and it has been shown that fatty acids act as mild disconnectors responsible for changes in trans-membrane potential that could result in ROS production [42]. Moreover, as confirmed by the presented results, a high-sucrose diet results in the accumulation of an advanced glycation end product (AGE). Levi and Werman [43] demonstrated that the oxidative degradation of sucrose or glucose adducts results in enhanced ROS production. It has been shown that AGE is also able to increase NADPH oxidase activity [44] and that a high-sucrose diet alters heart antioxidant enzyme activity [6] which, with the excessive production of ROS, leads to an imbalance of a redox status and accelerates oxidative stress.

The oral cavity is subjected to various external factors that have oxidizing potential and the ability to generate ROS. It is also known that salivary antioxidants and—to a lesser extent—plasma antioxidants constitute an important part of the oral antioxidant barrier. One of the key salivary antioxidants is salivary peroxidase which, together with catalase, neutralizes hydrogen peroxide (H_2_O_2_) formed as a result of the dismutation reaction with the participation of superoxide dismutase. Blood-derived uric acid contributes to 40% of the total salivary antioxidant barrier [10].

In both salivary glands of rats fed a high-sucrose diet, we observed a significant increase in SOD specific activity, which was more likely a cellular compensatory response to intensified superoxide anion generation than an attempt to regulate oxidative stress resistance. Tsang et al. [45] proved that a raised level of superoxide anions leads to an increase in superoxide dismutase activity and its relocation to the cell nucleus. The enzyme regulates the expression of protein genes responsible for oxidative stress resistance as well as protein genes responsible for repairing oxidative damage. Excess H_2_O_2_ formed through SOD-catalyzed dismutation of superoxide does not seem to be sufficiently neutralized because we did not observe an adequate response from the enzymes responsible for its neutralization (parotid glands of HSD rats: ↓ Px, CAT unchanged, submandibular glands of HSD rats: CAT and Px activities unchanged). Although it is known that H_2_O_2_ is not a free radical, it is able to cross the cell membranes and diffuse into different cellular compartments. Hydrogen peroxide that is not neutralized reacts very easily with transition metals and forms the highly reactive hydroxyl radical (OH^•^) responsible for the majority of oxidative damage in biological compartments [46]. We cannot explicitly explain the behavior of enzymes involved in the neutralization of hydrogen peroxide solely on the basis of the performed assays. Decreased as well as unchanged enzyme activity may result from the oxidative modification of polypeptide chains or the participation of enzymes in combating free radicals during the eight weeks of the diet. The impaired antioxidant defense may be also caused by non-enzymatic glycation of these enzymes in the course of hyperglycemia, which explains the negative correlation between Px activity in the parotid glands of HSD rats and plasma glucose concentration [47]. Clearly, the efficiency of the entire system cannot be assessed based on the analysis of only three antioxidant enzymes. However, one of the evaluated parameters was also the total antioxidant capacity, which is the sum of all antioxidants tested in the biological material (TAS). A significant decrease in TAS in the parotid glands, unchanged TAS in the submandibular glands of HSD rats, and the observed enhancement of oxidative modification in both salivary glands in the course of a high-sucrose feeding confirm the inefficiency of antioxidant systems of these glands in the eradication of ROS.

The results of our study revealed a greater-percentage increase in the concentration and diversity of oxidative damage as well as the intensity of OS to the parotid glands compared to the submandibular glands of rats fed a high-sucrose diet. In the parotid glands of the HSD rats, we observed a significant increase in all the assessed parameters of oxidative stress. In the submandibular glands of these rats, only the PC and 8-isoP levels were raised. It should also be emphasized that increased concentrations of oxidation products in the submandibular glands (PC ↑ 57%, 8-isoP ↑ 139%) were less pronounced than in the case of the parotid glands (PC ↑ 82%, 8-isoP ↑ 410%) of the HSD group vs. the controls. The above results confirm that the parotid glands are subjected to more intense oxidative stress or they are more susceptible to an oxidant attack generated in the course of the high-sucrose diet than the submandibular glands. On the other hand, the lower intensity and size of oxidative damage observed in the submandibular glands may indicate the initial stage of oxidative stress development.

The systemic oxidative stress-induced plasma redox balance changed, but blood composition did not seem to reflect salivary glands composition. However, we noticed the positive correlation between PC and AOPP concentration in the parotid glands and plasma HOMA-IR of the HSD group, which suggests that only the level of parotid protein peroxidation products increases as a function of insulin resistance while parotid lipid peroxidation, 8-OHdG, the submandibular lipid, and protein peroxidation products are independent of insulin sensitivity. We also demonstrated a positive correlation between plasma insulin and PC concentrations in the submandibular glands of the HSD rats. These results could be explained by the assumption that hyperinsulinemia leads to insufficient scavenging of hydrogen peroxide that is responsible for the oxidation of protein thiol groups in the cell [48].

We observed that the weight of the parotid glands of HSD rats was increased while the submandibular gland weight did not change. In addition, the high-sucrose diet was associated with severe morphological changes in the parotid glands and a small degree of vacuolization of the submandibular glands. These morphological changes appeared to be of a lipid nature since they had been removed during fixation and processing of the sample. It was also demonstrated that the consumption of a high-sucrose diet leads to an increase in tissue triglyceride accumulation [49]. Such severity of fatty degeneration in the parotid vs. submandibular glands of the HSD group appears to be the cause of intensified oxidative modifications in the parotid glands compared to the submandibular glands of HSD rats. It has been shown that adipose tissue increases the expression of proinflammatory monocyte chemoattractant protein-1 (MCP-1), which promotes macrophage infiltration and activates the inflammatory signaling pathway in adipocytes and macrophages [50]. This condition results in an increase in the secretion of pro-inflammatory cytokines and an increase in the production of ROS [23], which was confirmed by the presented study (↑ IL-1β, ↑ NADPH oxidase, ↑ ROS). It should be emphasized that NADPH oxidase is the main source of ROS in the cell and plays a critical role in the inflammatory processes [30,51]. 

We observed no changes in the volume of unstimulated whole saliva secretion, but the stimulated whole saliva as well as protein concentration and amylase activity in both glands were significantly reduced. It is believed that stimulated saliva is produced mainly by the parotid glands [52]. The impairment of the secretory function of the parotid glands may be explained by severe fatty degeneration, which reduces the active surface of secretory glands and leads to the destruction of residual glandular elements [53]. A decreased response of residual acinar cells to the muscarinic agonist can also be explained by the negative correlation between 4-HNE protein adducts in the parotid glands and a stimulated salivary flow of HSD rats. Evidence showed that the level of 4-HNE protein adducts increases the expression of proinflammatory cytokines and metalloproteinase production in the course of mitochondrial ROS-mediated stimulation of Akt/NF-kappaB signaling pathways. The cytokines and metalloproteinases have been shown to disturb the stromal tissue by decreasing the number and size of fibers, focal dilatations of axons, and fiber degeneration [54] and by blocking the interaction between the neurotransmitter and its receptor in the salivary glands of diabetic rats [55]. The latter mechanisms have also been described as the cause of a sympathetic system dysfunction in the salivary glands of diabetic rats [56]. Because the activity of salivary α-amylase is considered a marker of the sympathetic nervous system activity, we can assume that HSD rats presented lower activity of this branch of the autonomic nervous system in both glands [56]. This may be the reason for the observed decrease in salivary amylase activity and protein concentration in both salivary glands. On the other hand, it has been shown that impaired amylase secretion may result from insulin resistance, i.e., reduced sensitivity of target tissues to insulins and no anabolic effect of this hormone [57].

It should be recalled that insulin resistance is a pre-diabetic metabolic state. In these conditions, the significant decrease in the salivary gland function was confirmed in many previous clinical studies [17,58,59,60]. Moreover, the salivary gland dysfunction has been associated with higher prevalence of dental caries [17,60], oral candidiasis [61], periodontitis [58], mucous injury, and xerostomia [62].

In summary, we demonstrated redox imbalance in the salivary glands of rats fed the HSD diet and greater sensitivity of the parotid glands to oxidative cellular damage. We found that a high-sugar diet also disturbs the secretory function of the salivary glands, which—in addition to the cariogenic effect—may significantly impair the homeostasis of the oral cavity system.

Analyzing the results of our study, it ought to be noted that only the kinetic studies can unequivocally confirm whether it is a high-sucrose diet or insulin resistance that causes the observed changes in the salivary glands. The other limitations of our experiment also included: the use of an animal model, duration of the experiment, and the evaluation of only some, although the most frequently used, biomarkers of redox homeostasis and oxidative stress. On the other hand, it should be emphasized that the analysis of the redox system (antioxidant barrier, parameters describing oxidative stress) and secretory function of the rat salivary glands in the course of a high-sucrose diet is novel and has an important clinical aspect.

## 5. Conclusions

- A high-sucrose diet disturbs the redox balance of the parotid and submandibular salivary glands. However, the parotid glands are more vulnerable to both antioxidant disturbances and oxidative damage.
- Mechanisms involved in the synthesis/secretion of proteins are hindered in both of the salivary glands of HSD rats as compared to the controls.
- A high-sucrose diet reduces the stimulated secretory capacity of salivary glands vs. the control.

## Figures and Tables

**Table 1:** General characteristics of the control and high-sucrose (HSD) fed rats.

	Control	HSD	*p*
Body weight (mg)	355.2 (311.5–415.0)	407.1 (367.6–449.2)	0.0046
Food intake (g/day)	18.97 (12.84–28.22)	20.73 (12.93–31.03)	0.7203
Energy intake (g)/per rat/per week	67.45(63.6–76.6)	78.8(63.33–85.1)	0.6300
PG weight (mg)	0.092 (0.081–0.11)	0.101 (0.092–0.128)	0.0447
SMG weight (mg)	0.305 (0.271–0.342)	0.31 (0.28–0.373)	0.0780
Glucose concentration (mg/dL)	142.5 (100.6–159.4)	172.0 (145.3–189.2)	0.0008
Insulin concentration (mU/mL)	0.178 (0.155–0.197)	0.2683 (0.192–0.355)	0.0159
HOMA-IR	2.704 (1.873–3.251)	18.272 (15.471–25.136)	<0.0001

Abbreviations: HOMA-IR, Homeostatic Model Assessment of Insulin Resistance, PG, parotid glands, SMG, submandibular glands.

**Table 2:** Differences between submandibular and parotid glands of the control rats.

	PG	SMG	*p*
Total protein (mg/mL)	3842 (3338–4773)	5118 (3574–6998)	0.0499
Amylase (μg/100 mg protein)	3.355 (2.917–4.635)	2.028 (1.787–2.948)	0.0159
UA (μg/100 mg protein)	2.991 (1.761–3.481)	2.981 (2.461–4.662)	0.5992
CAT (μmol H_2_O_2_/min/100 mg protein)	3.596 (2.134–5.290)	2.95 (1.839–5.163)	0.7209
SOD (U/100 mg protein)	1.477 (0.6850–2.235)	1.656 (1.188–2.258)	0.5737
Px (mU/100 mg protein)	2.791 (1.821–3.421)	6.682 (6.342–7.242)	0.0002
TAS (μmol/100 mg protein)	2.780 (2.590–2.912)	1.450 (0.853–2.010)	0.0021
AGE (fluorescence/100 mg protein)	149.6 (123.9–163.8)	131.7 (72.62–155.7)	0.1049
AOPP (μmol/100 mg protein)	2.467 (1.532–3.742)	1.854 (1.159–2.291)	0.0148
PC (nmol/100 mg protein)	0.3485 (0.2157–0.4675)	0.2774 (0.2422–0.3490)	0.3095
8-isoP (fg/100 mg protein)	24.78 (17.64–41.88)	8.713 (5.974–10.97)	0.0159
4-HNE (μg/100 mg protein)	0.9725 (0.5400–1.320)	0.521 (0.2100–0.7600)	0.0173
8-OHdG (fg/100 mg protein)	33.14 (23.51–38.87)	0.4047 (0.2439–0.4287)	0.0079
TOS (umol/100 mg protein)	0.1320 (0.086–0.152)	0.0120 (0.009–0.018)	0.0006
OSI	4.871 (3.060–5.736)	0.7600 (0.603–1.572)	0.0006
IL-1β (pg/100 mg protein)	29.41 (23.0–38.96)	13.95 (11.98–17.2)	0.0043
NADPH oxidase (nmol O_2_^−^/min/100 mg protein)	1061.0 (972.5–1514.0)	975.5 (867.6–1275.0)	0.1605
ROS production (nmol/100 mg protein)	128.4 (11.6–140.4)	91.61 (55.47–153.7)	0.0650

Abbreviations: 4-HNE, 4-hydroxynonenal protein adducts, 8-isoP, 8-isoprostanes, 8-OHdG, 8-hydroxy-2’-deoxyguanosine, AGE, advanced glycation end products, AOPP, advanced oxidation protein products, CAT, catalase, IL-1β, interleukin-1β, OSI, oxidative stress index, PC, protein carbonyls, PG, parotid glands, Px, salivary peroxidase, ROS, reactive oxygen species, SOD, superoxide dismutase-1, SMG, submandibular glands, TAS, total antioxidant status, TOS, total oxidant status, UA, uric acid.

**Table 3:** Differences between submandibular and parotid glands of high-sucrose diet (HSD) fed rats.

	PG	SMG	*p*
Total protein (μg/mL)	3246 (2929–3847)	3599 (3459–4632)	0.0207
Amylase (μg/100 mg protein)	0.4090 (0.2340–0.5423)	0.3668 (0.2772–1.177)	0.8413
UA (μg/100 mg protein)	1.851 (1.620–2.741)	2.351 (2.000–3.141)	0.0156
CAT (umol H_2_O_2_/min/100 mg protein)	2.440 (1.306–3.951)	3.799 (1.462–5.518)	0.1605
SOD (U/100 mg protein)	1.999 (1.595–2.467)	2.657 (1.503–3.210)	0.0499
Px (mU/100 mg protein)	1.5 (1.300–2.381)	6.472 (6.142–7.102)	0.0002
TAS (μmol/100 mg protein)	1.640 (1.110–1.920)	1.480 (0.850–2.100)	0.9015
AGE (fluorescence/100 mg protein)	184.4 (133.8–215.3)	146.2 (100.7–181.7)	0.0104
AOPP (μmol/100 mg protein)	3.367 (2.905–5.098)	1.352 (1.025–1.907)	0.0007
PC (nmol/100 mg protein)	0.6203 (0.4416–0.8595)	0.4408 (0.3141–0.5422)	0.0317
8-isoP (fg/100 mg protein)	126.5 (92.96–158.5)	20.88 (17.64–29.32)	0.0079
4-HNE (μg/100 mg protein)	1.608 (1.270–1.810)	0.64 (0.3100–0.9200)	0.0043
8-OHdG (fg/100 mg protein)	62.14 (50.74–88.58)	0.6352 (0.4009–0.7201)	0.0079
TOS (μmol/100 mg protein)	0.3100 (0.222–0.398)	0.0210 (0.018–0.032)	0.0006
OSI	16.850 (11.560–35.860)	1.295 (1.055–2.353)	0.0006
IL-1β (pg/100 mg protein)	52.0 (41.0–64.7)	23.8 (18.01–28.22)	0.0043
NADPH oxidase (nmol O_2_^−^/min/100 mg protein)	1556.0 (1071.0–1775.0)	1248.0 (963.4–1685.0)	0.0499
ROS production (nmol/100 mg protein)	175.9 (113.9–206.6)	139.0 (97.44–228.2)	0.0410

Abbreviations: 4-HNE, 4-hydroxynonenal protein adducts, 8-isoP, 8-isoprostanes, 8-OHdG, 8-hydroxy-2’-deoxyguanosine, AGE, advanced glycation end products, AOPP, advanced oxidation protein products, CAT, catalase, IL-1β, interleukin-1β, OSI, oxidative stress index, PC, protein carbonyls, PG, parotid glands, Px, salivary peroxidase, ROS, reactive oxygen species, SOD, superoxide dismutase-1, SMG, submandibular glands, TAS, total antioxidant status, TOS, total oxidant status, UA, uric acid.

## References

[1] Johnson R.K., Appel L.J., Brands M., Howard B.V., Lefevre M., Lustig R.H., Sacks F., Steffen L.M., Wylie-Rosett J. (2009). Dietary sugars intake and cardiovascular health: A scientific statement from the american heart association. Circulation.

[2] WHO (1985). Energy and Protein Requirements. Report of a joint FAO/WHO/UNU Expert Consultation.

[3] WHO (2015). Sugar Intake for Adults and Children.

[4] Blouet C., Mariotti F., Azzout-Marniche D., Mathé V., Mikogami T., Tomé D., Huneau J.-F. (2007). Dietary cysteine alleviates sucrose-induced oxidative stress and insulin resistance. Free Radic. Biol. Med..

[5] Bonnard C., Durand A., Peyrol S., Chanseaume E., Chauvin M.-A., Morio B., Vidal H., Rieusset J. (2008). Mitochondrial dysfunction results from oxidative stress in the skeletal muscle of diet-induced insulin-resistant mice. J. Clin. Investig..

[6] Busserolles J., Zimowska W., Rock E., Rayssiguier Y., Mazur A. (2002). Rats fed a high sucrose diet have altered heart antioxidant enzyme activity and gene expression. Life Sci..

[7] Diniz Y.S., Rocha K.K.H.R., Souza G.A., Galhardi C.M., Ebaid G.M.X., Rodrigues H.G., Novelli Filho J.L.V.B., Cicogna A.C., Novelli E.L.B. (2006). Effects of n-acetylcysteine on sucrose-rich diet-induced hyperglycaemia, dyslipidemia and oxidative stress in rats. Eur. J. Pharmacol..

[8] Feillet-Coudray C., Sutra T., Fouret G., Ramos J., Wrutniak-Cabello C., Cabello G., Cristol J.P., Coudray C. (2009). Oxidative stress in rats fed a high-fat high-sucrose diet and preventive effect of polyphenols: Involvement of mitochondrial and nad(p)h oxidase systems. Free. Radic. Biol. Med..

[9] Zhou X., Han D., Xu R., Wu H., Qu C., Wang F., Wang X., Zhao Y. (2016). Glycine protects against high sucrose and high fat-induced non-alcoholic steatohepatitis in rats. Oncotarget.

[10] Knaś M., Maciejczyk M., Waszkiel D., Zalewska A. (2013). Oxidative stress and salivary antioxidants. Dent. Med. Probl..

[11] Maciejczyk M., Mikołuć B., Pietrucha B., Heropolitańska-Pliszka E., Pac M., Motkowski R., Car H. (2017). Oxidative stress, mitochondrial abnormalities and antioxidant defense in Ataxia-teleangiectasia, Bloom syndrome and Nijmegen breakage syndrome. Redox Biol..

[12] Gerdin E.W., Angbratt M., Aronsson K., Eriksson E., Johansson I. (2008). Dental caries and body mass index by socio-economic status in swedish children. Community Dent. Oral Epidemiol..

[13] Sheiham A., James W.P.T. (2014). A new understanding of the relationship between sugars, dental caries and fluoride use: Implications for limits on sugars consumption. Public Heal. Nutr..

[14] Tieche J.M., Leonora J., Steinman R.R. (1994). High sucrose diet inhibits basal secretion of intradentinal dye penetration-stimulating parotid hormone in pigs. J. App. Physiol..

[15] Tieche J.-M., Leonora J. (1995). Acute secretion of immunoreactive parotid hormone in response to different diets in the pig. Arch. Oral Biol..

[16] Zalewska A., Knaś M., Waszkiewicz N., Waszkiel D., Sierakowski S., Zwierz K. (2013). Rheumatoid arthritis patients with xerostomia have reduced production of key salivary constituents. Oral Surg. Oral Med. Oral Pathol..

[17] Zalewska A., Knaś M., Kuźmiuk A., Waszkiewicz N., Niczyporuk M., Waszkiel D., Zwierz K. (2013). Salivary innate defense system in type 1 diabetes mellitus in children with mixed and permanent dentition. Acta Odontol. Scand..

[18] Zalewska A., Knaś M., Gińdzieńska-Sieśkiewicz E., Waszkiewicz N., Klimiuk A., Litwin K., Sierakowski S., Waszkiel D. (2014). Salivary antioxidants in patients with systemic sclerosis. J. Oral Pathol. Med..

[19] Farley C., Cook J.A., Spar B.D., Austin T.M., Kowalski T.J. (2003). Meal Pattern Analysis Of Diet-Induced Obesity In Susceptible And Resistant Rats. Obes. Res..

[20] Kołodziej U., Maciejczyk M., Niklińska W., Waszkiel D., Żendzian-Piotrowska M., Żukowski P., Zalewska A. (2017). Chronic high-protein diet induces oxidative stress and alters the salivary gland function in rats. Arch. Oral Biol..

[21] Kołodziej U., Maciejczyk M., Miąsko A., Matczuk J., Knaś M., Żukowski P., Żendzian-Piotrowska M., Borys J., Zalewska A. (2017). Oxidative modification in the salivary glands of high fat-diet induced insulin resistant rats. Front. Physiol..

[22] Maciejczyk M., Kossakowska A., Szulimowska J., Klimiuk A., Knas M., Car H., Niklinska W., Ladny J.R., Chabowski A., Zalewska A. (2017). Lysosomal exoglycosidase profile and secretory function in the salivary glands of rats with streptozotocin-induced diabetes. J. Diabetes Res..

[23] Maciejczyk M., Żebrowska E., Zalewska A., Chabowski A. (2018). Redox balance, antioxidant defense, and oxidative damage in the hypothalamus and cerebral cortex of rats with high fat diet-induced insulin resistance. Oxid. Med. Cell Longev..

[24] Aebi H. (1984). Catalase in vitro. Methods Enzymol..

[25] Misra H.P., Fridovich I. (1972). The role of superoxide anion in the autoxidation of epinephrine and a simple assay for superoxide dismutase. J. Biol. Chem..

[26] Mansson-Rahemtulla B., Baldone D.C., Pruitt K.M., Rahemtulla F. (1986). Specific assays for peroxidases in human saliva. Arch. Oral Biol..

[27] Paglia D.E., Valentine W.N. (1967). Studies on the quantitative and qualitative characterization of erythrocyte glutathione peroxidase. J. Lab. Clin. Med..

[28] Erel O. (2004). A novel automated direct measurement method for total antioxidant capacity using a new generation, more stable ABTS radical cation. Clin. Biochem..

[29] Kalousová M., Skrha J., Zima T. (2002). Advanced glycation end-products and advanced oxidation protein products in patients with diabetes mellitus. Physiol. Res..

[30] Maciejczyk M., Szulimowska J., Skutnik A., Taranta-Janusz K., Wasilewska A., Wisniewska N., Zalewska A. (2018). Salivary Biomarkers of Oxidative Stress in Children with Chronic Kidney Disease. J. Clin. Med..

[31] Reznick A.Z., Packer L. (1994). Oxidative damage to proteins: Spectrophotometric method for carbonyl assay. Methods Enzymol..

[32] Erel O. (2005). A new automated colorimetric method for measuring total oxidant status. Clin. Biochem..

[33] Borys J., Maciejczyk M., Krętowski J., Antonowicz B., Ratajczak-Wrona W., Jabłońska E., Załęski P., Waszkiel D., Ładny J.R., Żukowski P. (2017). The redox balance in erythrocytes, plasma and periosteum of patients with titanium fixation of the jaw. Front. Physiol..

[34] Griendling K.K., Minieri C.A., Ollerenshaw J.D., Alexander R.W. (1994). Angiotensin II stimulates NADH and NADPH oxidase activity in cultured vascular smooth muscle cells. Circ. Res..

[35] Bondy S.C., Guo S.X. (1994). Effect of ethanol treatment on indices of cumulative oxidative stress. Eur. J. Pharmacol..

[36] Haber C.A., Lam T.K., Yu Z., Gupta N., Goh T., Bogdanovic E., Giacca A., Fantus I.G. (2003). N-acetylcysteine and taurine prevent hyperglycemia-induced insulin resistance in vivo: Possible role of oxidative stress. Am. J. Physiol. Endocrinol. Metab..

[37] Stentz F.B., Brewer A., Wan J., Garber C., Daniels B., Sands C., Kitabchi A.E. (2016). Remission of pre-diabetes to normal glucose tolerance in obese adults with high protein versus high carbohydrate diet: Randomized control trial. BMJ Open Diabetes Res. Care.

[38] Suwannaphet W., Meeprom A., Yibchok-Anun S., Adisakwattana S. (2010). Preventive effect of grape seed extract against high-fructose diet-induced insulin resistance and oxidative stress in rats. Food Chem. Toxicol..

[39] Ebertz C.E., Bonfleur M.L., Bertasso I.M., Mendes M.C., Lubaczeuski C., Araujo A.C., Paes A.M., Amorim E.M.P., Balbo S.L. (2014). Duodenal jejunal bypass attenuates non-alcoholic fatty liver disease in western diet-obese rats. Acta Cir. Bras..

[40] Gan K.X., Wang C., Chen J.H., Zhu C.J., Song G.Y. (2013). Mitofusin-2 ameliorates high-fat diet- induced insulin resistance in liver of rats. World J. Gastroenterol..

[41] Ogawa F., Shimizu K., Muroi E., Haseqawa M., Takehara M., Sato S. (2006). Serum levels of 8-isoprostane, a marker of oxidative stress, are elevated in patients with systemic sclerosis. Rheumatology.

[42] Cocco T., Di Paola M., Papa S., Lorusso M. (1999). Arachidonic acid interaction with the mitochondrial electron transport chain promotes reactive oxygen species generation. Free Radic. Biol. Med..

[43] Levi B., Werman M.J. (1998). Long-term fructose consumption accelerates glycation and several age-related variables in male rats. J. Nutr..

[44] Basta G., Schmidt A.M., De Caterina R. (2004). Advanced glycation end products and vascular inflammation: Implications for accelerated atherosclerosis in diabetes. Cardiovasc. Res..

[45] Tsang C.K., Liu Y., Thomas J., Zhang Y., Zheng X.F. (2014). Superoxide dismutase 1 acts as a nuclear transcription factor to regulate oxidative stress resistance. Nat. Commun..

[46] Lushchak V.L. (2014). Free radicals, reactive oxygen species, oxidative stress and its classification. Chem. Biol. Interact..

[47] Erejuwa O.O., Gurtu S., Sulaiman S.A., Ab Wahab M.S., Sirajudeen K.N., Salleh M.S. (2010). Hypoglycemic and antioxidant effects of honey supplementation in streptozotocin-induced diabetic rats. Int. J. Vitam. Nutr. Res..

[48] Rhee S.G. (2006). Cell signaling. H_2_O_2_, a necessary evil for cell signaling. Science.

[49] Robert L., Narcy A., Rayssiguier Y., Mazur A., Remesy C. (2008). Lipid metabolism and antioxidant status in sucrose vs. potato-fed rats. J. Am. Coll. Nutr..

[50] Solinas G., Karin M. (2010). JNK1 and IKKbeta: Molecular links between obesity and metabolic dysfunction. FASEB J..

[51] Kim H. (2008). Cerulein pancreatitis: Oxidative stress, inflammation, and apoptosis. Gut Liver.

[52] Choromańska M., Klimiuk A., Kostecka-Sochoń P., Wilczyńska K., Kwiatkowski M., Okuniewska N., Waszkiewicz N., Zalewska A., Maciejczyk M. (2017). Antioxidant defence, oxidative stress and oxidative damage in saliva, plasma and erythrocytes of dementia patients. Can salivary AGE be a marker of dementia?. Int. J. Mol. Sci..

[53] Żukowski P., Maciejczyk M., Matczuk J., Kurek K., Waszkiel D., Żendzian-Piotrowska M., Zalewska A. (2018). Effect of N-acetylcysteine on antioxidant defense, oxidative modification, and salivary gland function in a rat model of insulin resistance. Oxid. Med. Cell Longev..

[54] Perez P., Kwon Y.J., Alliende C., Leyton L., Aguilera S., Molina C., Labra C., Julio M., Leyton C., Gonzalez M.J. (2005). Increased acinar damage of salivary glands of patients with Sjogren’s syndrome is paralleled by simultaneous imbalance of matrix metalloproteinase 3/tissue inhibitor of metalloproteinases 1 and matrix metalloproteinase 9/tissue inhibitor of metalloproteinases 1 ratios. Arthritis Rheum..

[55] Anderson L.C., Garrett J.R., Thulin A., Proctor G.B. (1989). Effects of streptozocin-induced diabetes on sympathetic and parasympathetic stimulation of parotid salivary gland function in rats. Diabetes.

[56] Busch L., Sterin-Borda L., Borda E. (2002). Differences in the regulatory mechanism of amylase release by rat parotid and submandibular glands. Arch. Oral Biol..

[57] Morris P.A., Prout R.E.S., Proctor G.B., Garrett J.R., Anderson L.C. (1992). Lipid analysis of the major salivary glands in streptozotocin-diabetic rats and the effects of insulin treatment. Arch. Oral Biol..

[58] Aren G., Sepet E., Ozdemir D., Dinccag N., Guvener B., Firatli E. (2003). Periodontal health, salivary status, and metabolic control in children with type 1 diabetes mellitus. J. Periodontol..

[59] Lopez M.E., Colloca M.E., Paez R.G., Schallmach J.N., Koss M.A., Chervonagura A. (2003). Salivary characteristics of diabetic children. Braz. Dent. J..

[60] Siudikiene J., Machiulskiene V., Nyvad B., Tenovuo J., Nedzelskiene I. (2008). Dental caries increments and related factors in children with type 1 diabetes mellitus. Caries Res..

[61] Aitken-Saavedra J., Lund R.G., Gonzalez J., Huenchunao R., Perez-Vallespir I., Morales-Bozo I., Urzua B., Tarquinio S.C., Maturana-Ramirez A., Martos J. (2018). Diversity, frequency and antifungal resistance of Candida species in patients with type 2 diabetes mellitus. Acta Odontol. Scand..

[62] Navea Aguilera C., Guijarro de Armas M.G., Monereo Megias S., Merino Viveros M., Toran Ranero C. (2015). The relationship between xerostomia and diabetes mellitus: A little known complication. Endocrinol. Nutr..

